# DOES LONELINESS MEDIATE THE RELATIONSHIP BETWEEN EARLY-LIFE ADVERSITY AND COGNITIVE FUNCTION IN LATER LIFE?

**DOI:** 10.1093/geroni/igae098.4166

**Published:** 2024-12-31

**Authors:** Jinnan Ren, Kenneth Ferraro

**Affiliations:** Purdue University - West Lafayette, West Lafayette, Indiana, United States; Purdue University, West Lafayette, Indiana, United States

## Abstract

Research has consistently linked negative childhood experiences to cognitive decline in later life, but the mechanisms that may mediate this relationship remain unclear. This study explores the influence of early-life adversity (ELA) and loneliness on cognitive function in later life. We analyzed three waves of data (2006-2010) from the Health and Retirement Study (N=10,787) to compare the potential influence of an ELA composite and six distinct ELA domains (SES disadvantage, disruptive home environment, risky parental behavior, chronic disease, impairment, and risky adolescent behavior) on cognitive function in later life. In addition, we examined whether loneliness in later life mediates this relationship using structural equation modeling. We found that the ELA composite was not associated with a decline in cognition two years later. However, in terms of ELA domains, SES disadvantage, disruptive home environment, risky parental behavior, and impairment in childhood are each associated with cognitive function in later life. Further, results indicated that loneliness partially mediated the relationship between two specific ELA domains — (a) disruptive home environment and (b) risky parental behavior — and cognitive function. However, the direct effects of ELA composite and risky adolescent behavior on cognitive function were insignificant, indicating complete mediation by loneliness in these cases. This study demonstrates how loneliness mediates the relationship between certain early-life risk factors and later-life cognitive decline. It highlights the importance of policy interventions aimed at reducing harmful family environments and addressing adolescent behavior issues. Strategies to reduce loneliness in later life may protect cognitive function among older adults.

